# Low glycaemic index diets improve glucose tolerance and body weight in women with previous history of gestational diabetes: a six months randomized trial

**DOI:** 10.1186/1475-2891-12-68

**Published:** 2013-05-24

**Authors:** Sangeetha Shyam, Fatimah Arshad, Rohana Abdul Ghani, Norasyikin A Wahab, Nik Shanita Safii, Mohd Yusof Barakatun Nisak, Karuthan Chinna, Nor Azmi Kamaruddin

**Affiliations:** 1 School of Post Graduate Studies and Research, International Medical University, Kuala Lumpur, Malaysia; 2 Department of Nutrition and Dietetics, International Medical University, Kuala Lumpur, Malaysia; 3 Endocrine Unit, Department of Medicine, Faculty of Medicine, Universiti Kebangsaan Malaysia (National University of Malaysia), Kuala Lumpur, Malaysia; 4 Dietetics Program, School of Healthcare Sciences, Faculty of Health Sciences, Universiti Kebangsaan Malaysia, Kuala Lumpur, Malaysia; 5 Department of Nutrition & Dietetics, Faculty of Medicine & Health Sciences, Universiti Putra Malaysia, Serdang, Malaysia; 6 Epidemiology and Biostatistics Unit, Department of Social and Preventive Medicine, Faculty of Medicine, University of Malaya, Kuala Lumpur, Malaysia

**Keywords:** Gestational diabetes mellitus, Type 2 diabetes, Diabetes prevention, Glycaemic index, Glycaemic load, Diet, Randomized clinical trial, Carbohydrates

## Abstract

**Background:**

Gestational Diabetes Mellitus (GDM) increases risks for type 2 diabetes and weight management is recommended to reduce the risk. Conventional dietary recommendations (energy-restricted, low fat) have limited success in women with previous GDM. The effect of lowering Glycaemic Index (GI) in managing glycaemic variables and body weight in women post-GDM is unknown.

**Objective:**

To evaluate the effects of conventional dietary recommendations administered with and without additional low-GI education, in the management of glucose tolerance and body weight in Asian women with previous GDM.

**Method:**

Seventy seven Asian, non-diabetic women with previous GDM, between 20- 40y were randomised into Conventional healthy dietary recommendation (CHDR) and low GI (LGI) groups. CHDR received conventional dietary recommendations only (energy restricted, low in fat and refined sugars, high-fibre). LGI group received advice on lowering GI in addition. Fasting and 2-h post-load blood glucose after 75 g oral glucose tolerance test (2HPP) were measured at baseline and 6 months after intervention. Anthropometry and dietary intake were assessed at baseline, three and six months after intervention. The study is registered at the Malaysian National Medical Research Register (NMRR) with Research ID: 5183.

**Results:**

After 6 months, significant reductions in body weight, BMI and waist-to-hip ratio were observed only in LGI group (P<0.05). Mean BMI changes were significantly different between groups (LGI vs. CHDR: -0.6 vs. 0 kg/m^2^, P= 0.03). More subjects achieved weight loss ≥5% in LGI compared to CHDR group (33% vs. 8%, P=0.01). Changes in 2HPP were significantly different between groups (LGI vs. CHDR: median (IQR): -0.2(2.8) vs. +0.8 (2.0) mmol/L, P=0.025). Subjects with baseline fasting insulin≥2 μIU/ml had greater 2HPP reductions in LGI group compared to those in the CHDR group (−1.9±0.42 vs. +1.31±1.4 mmol/L, P<0.001). After 6 months, LGI group diets showed significantly lower GI (57±5 vs. 64±6, P<0.001), GL (122±33 vs. 142±35, P=0.04) and higher fibre content (17±4 vs.13±4 g, P<0.001). Caloric intakes were comparable between groups.

**Conclusion:**

In women post-GDM, lowering GI of healthy diets resulted in significant improvements in glucose tolerance and body weight reduction as compared to conventional low-fat diets with similar energy prescription.

## Background

History of gestational diabetes mellitus (GDM) is a non-modifiable risk factor for developing type 2 diabetes mellitus (T2DM) [1,2]. Frequently, women with prior GDM exhibit tendencies for central obesity, insulin resistance and glucose intolerance [3]. Thirty-three to 50% of GDM women develop overt T2DM five years after delivery [4]. At ten years postpartum, 35–60% of GDM women are known to develop type 2 DM [3]. Hence GDM represents a challenging group to institute effective intervention [5].

Intensive lifestyle intervention has shown to be effective in reducing risk for T2DM in high risk individuals [6,7]. Conventional diets for primary prevention of T2DM consistently support energy-restricted, low-fat, and high–complex carbohydrate regime to achieve weight loss [8,9]. However, such interventions have shown limited success in reducing postpartum weight in women after GDM [10].

Meanwhile, emerging evidence emphasizes the role of insulin secretion and insulin resistance in body weight regulation [11]. Therefore, dietary factors that influence these parameters would include the type of carbohydrate defined by its glycaemic index (GI) and its overall glycaemic potential described by its glycaemic load (GL, i.e. sum of the product of GI and available carbohydrate amounts for all foods consumed in the diet [12]) are known to modulate weight loss, especially in hyper-insulinaemic women [13,14]. Low GI diets also improve glycaemic control in Asian diabetics [15] and reduce risk for T2DM [16]. However the effect of lowering dietary GI in women with prior GDM is currently unknown. This study aimed to evaluate the effect of adding low GI dietary advice to conventional dietary prescription on glycaemic variables and body weight in comparison to conventional dietary prescription among women with previous GDM.

## Methods

This study was conducted at the endocrine clinic of a tertiary hospital. The project was approved by the Ethic and Research Review Committees of the institutions involved, in-line with national regulations and according to the International Harmonization Consensus on Good Clinical Practice. The study is registered at the Malaysian National Medical Research Register with Research ID: 5183. The trial was carried out according to Consolidated Standards of Reporting Trials (CONSORT) guidelines.

### Inclusion and exclusion criteria

Healthy women in the age group of 20–40 years old, with a history of GDM (defined according to WHO criteria, [17]), and high risks for developing T2DM were included. While GDM itself is a non-modifiable risk factor for T2DM, certain modifiable risk factors that increase the risk in this group of subjects have been identified. These include central obesity, higher postpartum body mass index (BMI), fasting blood sugar (FBS), 2 h post-load blood glucose (2HPP) and energy intake [18,19]. Hence preventive interventions would be especially beneficial to subjects who have high risks as indentified by the inclusion criteria of the study. We therefore defined high risk as satisfying one of the following four conditions: BMI>23 kg/m^2^, or waist circumference> 80 cm [20], or impaired glucose tolerance (IGT) defined as 2HPP after 75 g screening oral glucose tolerance test (OGTT) ≥7.8 and<11.1 mmol/L or impaired fasting glucose (IFG) defined as FBS>5.6 mmol/L or a family history of T2DM. Exclusion criteria were the diagnosis of T2DM (FBS≥7.0 mmol/L, or 2HPP≥11.1 mmol/l, [17], or presence of other health complications and usage of drugs affecting body weight and glucose control. Written informed consent was obtained from all participants. The women were enrolled into the trial at a minimum of two months after their last GDM delivery.

### Measurement of outcome variables

The primary end point of this study was 2HPP after a 75 g oral glucose tolerance test was administered. FBS, fasting serum insulin (INS) and anthropometric measures were the secondary endpoints investigated.

Body weight rounded to 0.1 kg was measured in light clothing, after emptying pockets and without footwear, using digital weighing scales (Model: BWB-800A, Tanita Corporation, Tokyo, Japan). Height without footwear was measured using the wall mounted stadiometer (Model No. 206, SECA, Hamburg, Germany), rounded to 0.1 cm to calculate BMI. BMI was calculated as the ratio of weight in kg to the square of height in m^2^. Waist and hip circumference in cm was measured as per WHO guidelines [21] and waist-to-hip ratio was calculated. All anthropometric measurements were done by a single researcher to avoid bias. Anthropometric measurements were made at baseline, three and six months after intervention.

Blood samples were collected after a 12-h fasting, and analysed for FBS and insulin. A 75 g OGTT was administered and 2HPP samples were obtained. Blood glucose was analysed using the G6PD/Hexokinase method as per routine medical centre laboratory protocol. Serum samples for INS were frozen at −20°C until batch processed for analysis. INS was analysed with chemiluminescent enzyme-labelled immunometric assay, using Immulite 2000 automated analyser (Diagnostic Products Corporation, Los Angeles, USA). Blood glucose and INS values were measured at baseline and 6 months after intervention.

Dietary intake was assessed with 3-day dietary records. The subjects were asked to report intake for any 2 weekdays and one weekend day for each visit. Subjects were trained to use the diet records at the screening visit and pictures of household measures were provided to assist the subjects with recording the amounts. Diet records were collected at baseline, three and six months after intervention. All food records were reviewed with subjects during their follow up visits by the research nutritionist to ensure completeness of entries.

GL by definition is the “product of the GI of foods and its carbohydrate content” [22]. GL for individual food items in the dietary record was calculated as the product of diet GI and carbohydrate intake divided by 100 [23]. Dietary GL represents both the quantity and the quality of carbohydrate in diet [22] and was calculated as sum total of GL of foods consumed in the day [24]. Diet GI was calculated using the formula Diet GI= Diet GL×100/amount of carbohydrate in the diet [24].

A Microsoft Excel-based food composition database and diet intake calculator- “DietPLUS Version 3” was built for this study and was used for dietary analysis [25]. Due to lack of availability of local GI values, GI values for numerous Malaysian foods needed to be matched or estimated. Hence a systematic GI value assignment to foods in the Malaysian food composition table to similar foods published internationally was undertaken. To improve the accuracy of this GI-matching process, the GI assignment was based on previously published methodology [26]. The international GI and GL database and its updated version the online database of University of Sydney was used for this purpose [27,28]. The methodology of adding glycaemic index and glycaemic load functionality to the Malaysian food composition database has been previously published [25].

International Physical activity short Questionnaire (IPAQ-short) was used to assess physical activity levels of the subjects [29].

### Randomisation

All eligible subjects (n=77) were randomized strictly according to a computerized allocation (1:1) list generated using randomisation software [30]; by an individual from outside this research group. The researchers were unaware of the sizes of blocks used. Participants were randomized to either a conventional healthy dietary recommendation (CHDR) or the “CHDR+Low GI” group (LGI).

### Dietary intervention

Details on the educational intervention and tools used have been published earlier [31]. However for the sake of completeness a brief description is provided here. The aim of the nutrition education was to achieve and maintain a 5-7% reduction in body weight if BMI>23 and maintain current weight if BMI<23 during the one year complete trial period. The nutrition intervention aimed to achieve this objective through establishing two diets that were similar in energy and macronutrient content but with different dietary GI. Individual energy requirement was calculated by multiplying basal metabolic rate (BMR) by an appropriate activity factor [32] based on reported physical activity pattern and occupation. Harris Benedict method was used to calculate BMR [32]. If the subject had a BMI>23 and if she was not breast feeding, the energy prescription was reduced by 500 Kcal. Subjects with BMI< 23 or those breastfeeding an infant<6 mo of age were prescribed their energy requirement (energy prescribed=energy requirement). All EP was rounded to the nearest hundred. Energy prescription was capped at 1800 Kcal/day. Each subject was given an individualized dietary sheet with the recommended number of servings of each food group per day according to their energy prescription. Subjects were taught meal planning based on their individualized energy prescription and a sample one day menu was provided during the intervention visit. Concepts of serving size, number of servings and food exchange groups were taught to both groups of subjects. These dietary prescriptions were indicative in nature as the study subjects were “out-patients” and intervention was educational in nature.

Primary nutrition educational intervention was administered through a structured one-to-one session with the research nutritionist, at baseline. Conceptual guidance to achieve conventional recommended diet (CHDR) low in fat and refined sugars, and high in fibre was provided using the 5M framework proposed by the Malaysian Ministry of Health ( minimize salt, sugar, oil, more fruit and vegetables) [33]. Subjects were encouraged to indulge in moderate physical activity for 30min, at least five times a week.

The LGI group in addition to the above conventional recommendations also received a GI-education component that taught subjects to substitute high GI foods with low GI options. Staple foods primarily determine dietary GI [34], hence, GI-education focused on the strategies to select low-GI options for high GI rice, bread and breakfast cereals. Subjects were advised to restrict rice intake to once per day since most locally available rice varieties were high in GI [35]. They were encouraged to choose lower GI foods like spaghetti, noodles or multi-grain bread at other times. Subjects were not required to memorize numerical GI values of foods. However a list of foods that classified foods as high, moderate or low GI was provided to aid making choices. The LGI subjects were asked to include one low GI food at each meal.

Colour coded (pink for LGI and blue for CHDR) take-home booklets were also provided to the subjects as added reference. To improve retention of participants in the trial two electronic interactions (short messaging service (SMS) or e-mail, as per the subject’s preference) per month was established. These contacts were similar between the groups in terms of frequency and content of delivery.

### Sample size calculation

Sample size was calculated to detect at the end of one year, a 1.5 mmol/L difference in the postprandial blood glucose, the primary end point for this study. This difference is thought to be of clinical significance [36] between the diet treatments, with a power of 80%. This was based on the assumption that the standard deviation of the response variable is 1.5 mmol/L [37]. A total of 34 subjects, 17 per group, were required for this study. Taking into account the higher attrition rates in preventive trials [38], and reported reluctance among women post-GDM to participate [5], we started with a bigger sample size.

### Statistical analysis

Statistical analyses were performed using IBM SPSS (Version 19, Somers, NY, USA). The effect of the respective dietary interventions on anthropometric measurements and glycaemic variables at the end of 6 months was investigated. Primary analysis included intent to treat analysis of anthropometric data and glycaemic variables. Missing data was imputed conservatively with the last observation carried forward technique. A secondary analysis was carried out using the completers’ analysis. For the dietary data analysis the baseline values and a mean of the intake at three and six months visit was used. A sub-analysis to determine the differential response to the dietary intervention in subjects with varying fasting insulin levels was carried out. Fasting insulin value of 2 μIU/ml was chosen to be the cut-off for this analysis since it was approximately the natural median INS value for our subjects

The statistical significance standard was set at 5%. Data normality was tested using the Shapiro–Wilks test. Data is presented as Mean ± SD, unless stated otherwise. Differences between groups were assessed using independent samples *t* test and changes in outcomes before and after intervention were analysed using paired *t* tests. When the test assumptions were not met, Mann–Whitney U-test test for two samples was used to compare values between groups and Wilcoxon Sign test was used to compare the changes in outcome within the group. Effect size values were computed to compare the effects of the diet treatments [14]. Effect sizes between 0.2-0.5, 0.5-0.8 and >0.8 were taken to denote “small”, ”moderate” and “large” changes in outcomes [14].

Linear regression analysis was used to study the role of energy intake, macronutrient intakes, dietary fibre, GI and GL as predictor variables on changes in body weight. In modelling 2HPP, in addition to the above mentioned predictors (dietary variables), change in body weight was included. In modelling FBS, in addition to dietary variables mentioned above, changes in body weight and 2HPP were included. A sub-analysis was done grouping subjects with INS<2 μIU/L and ≥2 μIU/L in studying the models as attempted above. We also wanted to test if the effect of GI on 2HPP was mediated by changes in weight, among the subjects with INS≥2 μIU/L. In this test the Sobel’s Method was used [39].

## Results

**Figure 1:**
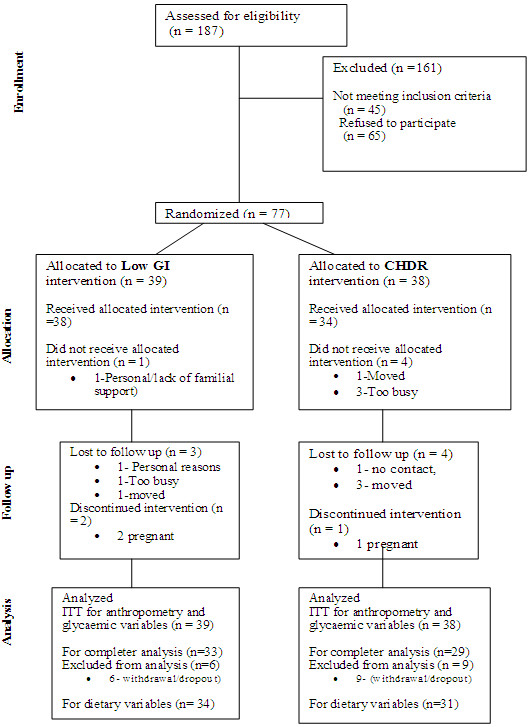
**Diagram of the flow of participants.** Legend: CHDR: Group receiving Conventional Healthy Dietary Recommendations Only: Low GI: Group receiving Conventional Healthy Dietary Recommendations +Low GI education, ITT: intent-to-treat analysis.

Seventy-seven subjects were recruited out of whom a total of 62 (29 in CHDR and 33 in LGI group) completed the 6 months on the trial (refer to Figure 1). The two groups had comparable baseline characteristics as shown in Table 1.

**Table 1:** Baseline characteristics of subjects (Mean±SD)

**Characteristic**	**LGI**	**CHDR**	**P**^**1 **^**Value**
Age (y)	30.9±4.3	31.5±4.5	0.999
No. of Pregnancies*	2±2	2±1	0.245
No. of GDM Pregnancies*	1±0	1±1	0.891
Parity*	2±2	2±2	0.671
Duration since Last GDM delivery (mo)*	6±3	5±2	0.246
Weight (kg)	65.3±11.5	64.6±12.5	0.794
BMI (kg/m^2^)	26.4±4.6	26.3±4.6	0.952
% subjects with BMI>23 (Overweight)	74.4	71.1	0.802
% subjects with BMI>25	59	63.2	0.816
% subjects with BMI>27.5 (Obese)	38.5	47.5	0.494
% subjects breast feeding	10.3	21.1	0.420
Waist Circumference (cm)	83.2±8.5	82.7±9.6	0.789
Waist: Hip Ratio	0.81±0.06	0.80±0.05	0.608
Fasting blood glucose(mmol/L)	4.7±0.54	4.8±0.53	0.405
2 h post 75 g OGTT blood glucose (mmol/L)	6.8±1.6	6.2±1.4	0.067

Legend: *LGI* Low GI Group, *CHDR* Conventional healthy Dietary Recommendation Group.

*Expressed as median±interquartile range, P^1^ Value: test between groups.

### Changes in anthropometry

Intent to treat (ITT) analysis showed significant reductions from baseline to six months in body weight (P=0.018), BMI (P=0.008), waist circumference (P<0.001) and waist-hip-ratio (P=0.02) in LGI group. In CHDR group there was a significant reduction in waist circumference (P=0.048) only (Refer Table 2). The effect sizes for the changes in weight, BMI, waist circumference and waist-hip-ratio from baseline to six months in the LGI group were 0.40, 0.45, 0.70 and 0.38, respectively which are moderate to large. The effect sizes for the changes in the same variables in the CHDR group were merely 0.02, 0.02, 0.33 and 0.22, respectively.

**Table 2:** Anthropometric outcomes during the course of the study (Mean±SD)

**Outcome**	**LGI**			**CHDR**		
	**baseline**	**3 months**	**6 months**	**baseline**	**3 months**	**6 months**
Weight (Kg)	65.3±11.5^a^	64.6±11.5^b^	64.0±11.7^ab^	64.6±12.5	64.7±12.6	64.5±13.0
BMI(kg/m2)	26.4±4.6^a^	26.0±4.7^a^	25.8±4.7^a^	26.3±4.6	26.4±4.7	26.3±4.8
WC (cm)	83.2±8.5 ^a^	83.0±10.4^b^	80.5±9.1^ab^	82.7±9.6 ^a^	82.5±10.8	81.5±10.1^a^
WHR	0.81±0.06^a^	0.80 ±0.06	0.79 ±0.05^a^	0.80±0.05	0.80±0.05	0.79±0.05

Legend: *LGI* Low GI Group, *CHDR* Conventional healthy Dietary Recommendation Group, *BMI* Body mass index, *WC* waist circumference *WHR* waist hip ratio.

For each group, values in a row with similar alphabetical superscripts show significantly different means in paired tests.

**Figure 2:**
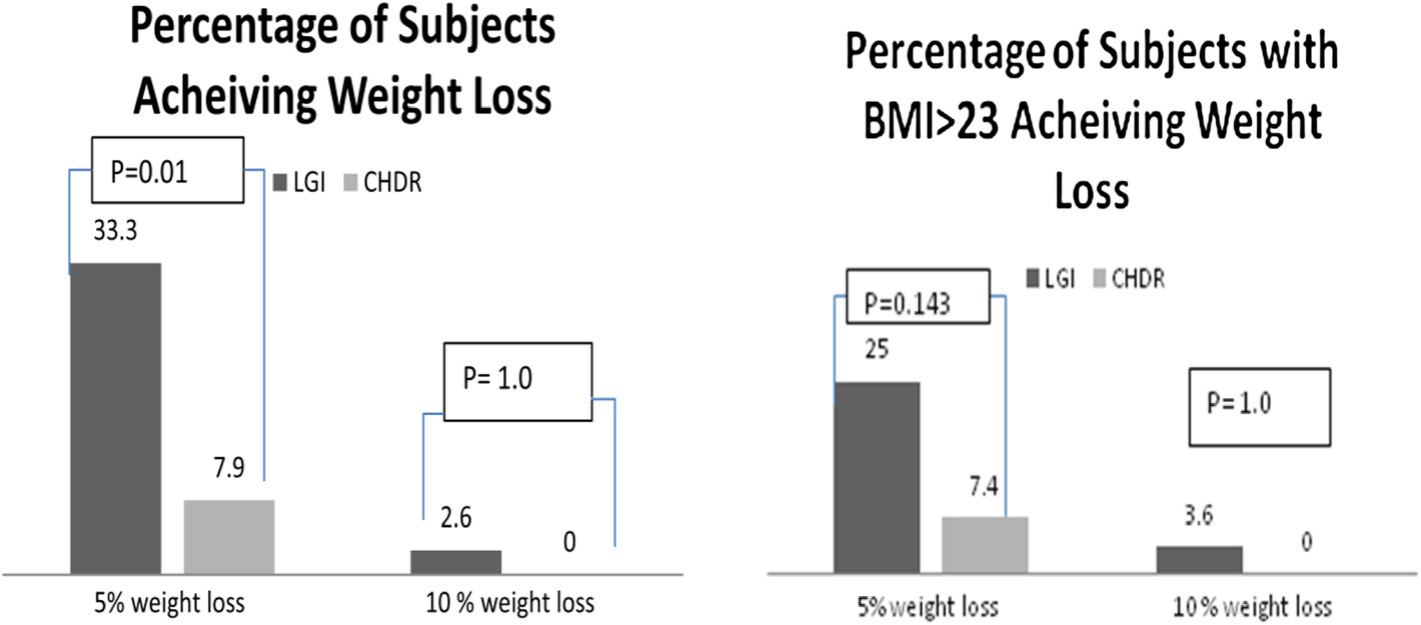
**Percentage weight loss in study groups.** Legend: LGI: Low GI Group, CHDR: Conventional healthy Dietary Recommendation Group, P value shown is calculated between the percentage of subjects in both groups using Fischer’s Exact test.

As shown in Figure 2, a greater number of subjects achieved a percentage weight loss≥5% in LGI as compared to CHDR Group (33% vs. 8%, P=0.01)

### Changes in glycaemic variables

The subjects had normoglycaemia or dysglycaemia (impaired fasting glucose/ impaired glucose tolerance) as tested by 75 g OGTT at baseline. FBS and 2HPP levels in the two groups were comparable at baseline (See, Table 3).

**Table 3:** Changes from baseline in glycaemic values (Mean±SD)

	**LGI**			**CHDR**		
	**Baseline**	**6 months**	**P**^**1**^	**Baseline**	**6 months**	**P**^**1**^
FBS (mmol/L)	4.7±0.53	5.0±1.2	0.060	4.8±0.53	4.9±0.53	0.111
∆FBS (mmol/L)*	−0. 2 (0.6)			0.1(0.6)		
2HPP (mmol/L)	6.8±1.6	6.8±2.8	0.960	6.2±1.4	6.8±1.6	0.010
∆2HPP (mmol/L)*	−0.2 (2.8) ^a^			0.8 (2.0)^a^		

Legend: *LGI* Low GI Group, *CHDR* Conventional healthy Dietary Recommendation Group. *FBS* Fasting blood sugar, *2HPP* 2 hour post load blood glucose after 75 g OGTT. P1: Significance of changes within groups.

* Data presented as median (interquartile range).

^a^ Difference between groups significant (P=0.025).

From baseline to 6 months, the changes in fasting blood sugar were not significant in both groups (See, Table 3). There was no significant change in median 2HPP in the LGI group (−0.2 (IQR: 2.8) mmol/L, P= 0.960). In the CHDR group, there was a significant increase in median 2HPP (+0.8 (IQR: 2.0) mmol/L, P=0.01). The changes in 2HPP were significantly different between groups (P=0.025, See, Table 3).

Results obtained from per-protocol analysis for changes in anthropometric and glycaemic outcomes were similar to the respective analysis done using ITT (data for per-protocol analysis not shown here).

### Baseline glucose tolerance status and response to dietary treatment

The anthropometric and glycaemic changes among subjects varying in baseline glucose tolerance status are shown in the following Table 4. There was a slight increase in weight and 2HPP blood glucose among subjects with dysglycaemia in CHDR group but in the LGI group there was a slight decrease in these two measurements. Among subjects with dysglycaemia at baseline 37.5% of subjects in CHDR (n= 3/8) and 64.3% in LGI (n=9/14) reverted to normoglycaemia (RR: 1.5, P= 0.378).

**Table 4:** Anthropometric and glycaemic changes in subjects with varying glucose tolerance status during the six months study period (Mean±SD)

**Baseline glucose tolerance status**	**Normoglycaemia**			**Dysglycaemic subjects**		
**Outcome changes**	**CHDR**	**LGI**	**P**	**CHDR**	**LGI**	**P**
**Weight (kg)**	−0.2±2.72	−1.93±3.48*	0.446	0.5±2.97	−0.7±3.37	0.446
**BMI (kg/m2)**	−0.08±1.04	−0.76±1.41*	0.077	0.25±1.2	−0.6±1.54	0.230
**WC (cm)**	−1.33±3.34	−3.72±4.24**	0.046	−2.14±4.53	−2.67±3.66*	0.786
**WHR**	−0.01±0.04	−0.02±0.005	0.427	−0.02±0.05	−0.03±0.03*	0.282
**FBS (mmol/L)**	0.15±0.41	0.31±0.53*	0.290	0.04±0.45	0.49±1.7	0.507
**2HPP (mmol/L)**	0.88±1.2***	0.24±2.04	0.216	0.47±2.22	−0.49±4.05	0.572

Legend: *LGI* Low GI Group, *CHDR* Conventional healthy Dietary Recommendation Group. *BMI* Body mass index, *WC* waist circumference, *WHR* waist hip ratio, *FBS* Fasting blood sugar, *2HPP* 2 hour post load blood glucose after 75g OGTT.

P values show the significance of difference in changes between groups.

Values with asterisks denote significant changes in paired-tests, *P≤0.05, **P≤0.01, ***P≤0.001.

### Fasting insulin levels and response to dietary treatment

Out of the 77 subjects, 44 (57.1%) had INS<2 μIU/L and 33 (42.9%) had INS≥2 μIU/L. The percentage of subjects with INS<2 μIU/L in the LGI and CHDR groups were 61.5 and 52.6% respectively. This difference between the groups was not significant (P=0.228).

Among the subjects with INS<2 μIU/L, there was no significant difference in the anthropometric and glycaemic changes for both LGI and CHDR groups (data not shown).

**Figure 3:**
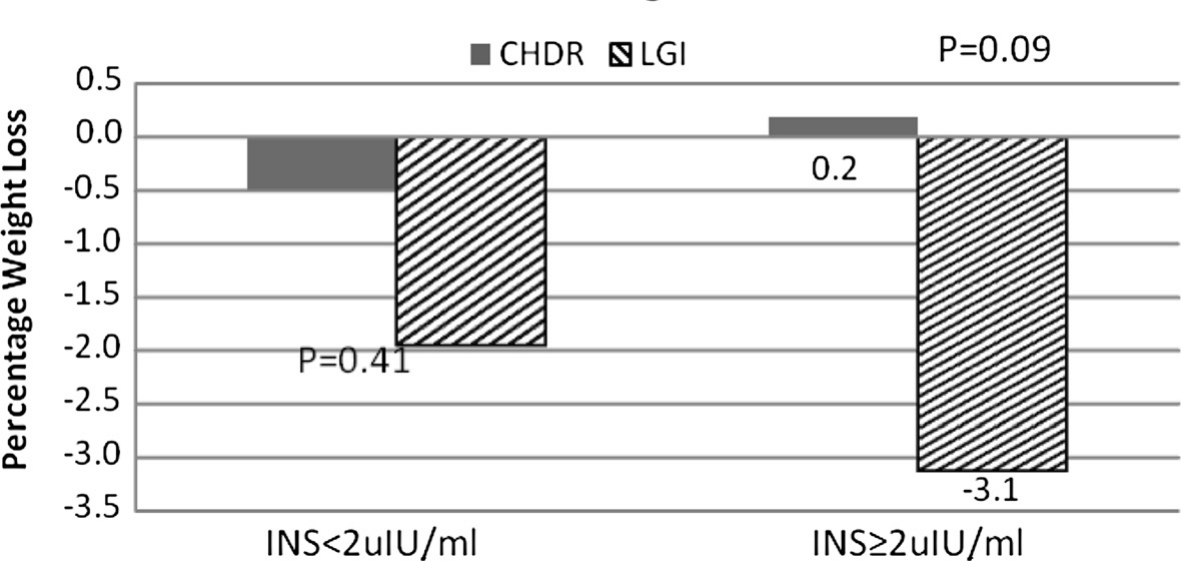
**Percentage weight loss among subjects categorized by baseline fasting insulin levels.** Legend: LGI: Low GI Group, CHDR: Conventional healthy Dietary Recommendation Group, P values for significance of changes between the diet groups are shown.

As shown in Figure 3, among subjects with baseline INS≥2 uIU/ml the percentage weight loss was higher in the LGI group compared to the CHDR group (−3.1% vs. + 0.2%, P=0.09).

**Figure 4:**
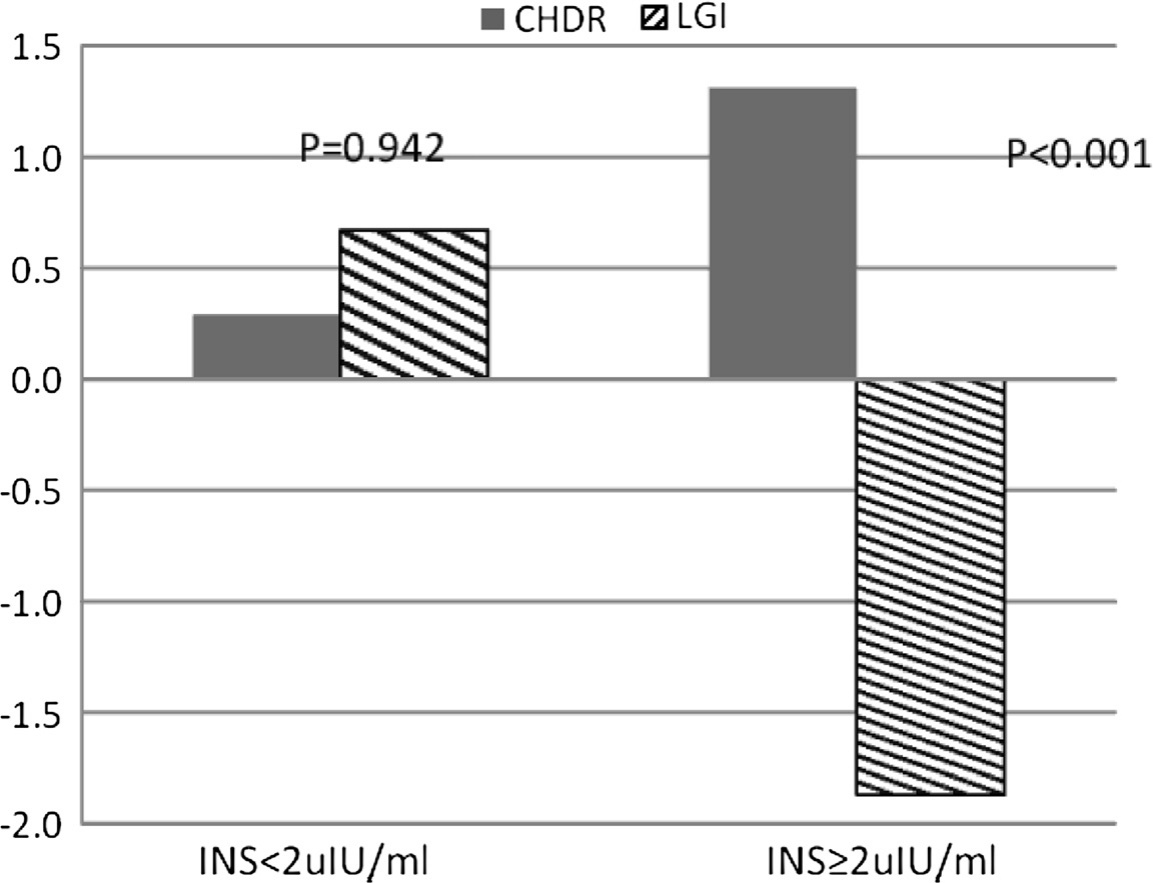
**2H Post-75g oral glucose load blood glucose changes among subjects categorized by baseline fasting insulin (mmol/L).** Legend: LGI: Low GI Group, CHDR: Conventional healthy Dietary Recommendation Group, P values for significance of changes between the diet groups are shown.

**Figure 5:**
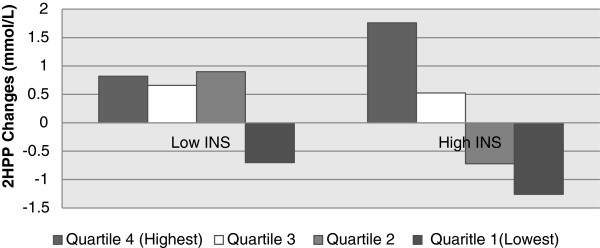
**Baseline fasting insulin levels and 2HPP changes among dietary GI quartiles.** Legend: Low INS: subjects with Baseline fasting INS < 2 uIU/L, High INS subjects with Baseline fasting INS ≥ 2 uIU/ml. Quartiles refer to dietary GI quartiles at 6 months.

For 2HPP, LGI subjects with baseline INS≥2 μIU/ml, recorded a significant reduction (−1.9±0.42 mmol/L, P=0.002) while the 2HPP levels increased (1.31± 1.4, P=0.004) among similar subjects in the CHDR group (See Figure 4). The level of 2HPP changes between the groups was significant (P <0.001). A sizable increase in 2HPP levels among subjects with baseline INS≥2 μIU/ml, was noted among those in the highest GI quartile (P value for trend =0.019, See Figure 5). There was no significant association between GI and changes in 2HPP among subjects with baseline INS<2 μIU/ml (P=0.576, See Figure 5).

Changes in FBS were however not significant within or between diet groups in such subjects (data not shown).

### Changes in dietary intake

Food records were collected from the subjects at baseline, at 3 months and at 6 months. Food records from 34 subjects in LGI group and 31 subjects in CHDR group were used in the analysis. The results of the dietary analysis are presented in Table 5. The after intervention values are the average between three and six month values.

**Table 5:** Dietary intake of subjects before and after intervention (Mean ± SD)

**Dietary outcome**	**LGI**			**CHDR**			**P values for difference in changes between groups**
	**Baseline**	**After intervention**	**P**^**1**^	**Baseline**	**After intervention**	**P**^**1**^	
Calories	1804±495	1660±368	0.075	1721± 491	1612±352	0.171	0.970
CHO g	245±75	206±52	0.002	225±68	220±48	0.627	0.224
CHO en%	55±9	51±5**	0.050	53±7	55±7**	0.240	0.065
Protein g	66±21	69±15	0.323	70±26	63±21	0.119	0.191
Protein en%	15±4	20±6	<0.001	16±5	19±7	0.124	0.400
Fat g	62±23	58±18	0.418	60±20	53±16	0.064	0.695
fat en%	29±8	28±5	0.368	30±6	25±7	0.004	0.177
Dietary Fibre (g)	13±5	17±4***	0.001	13±5	13±4***	0.583	0.022
Glycaemic Index	62±5	57±5***	0.003	62±7	64±6***	0.4	0.033
Glycaemic Load	152±41	122±33*	<0.001	142±42	141±35*	0.854	0.085

*LGI* Low GI Group, *CHDR* Conventional Healthy Dietary Recommendation Group.

P^1-^ P values for changes within groups.

*Values significantly different between groups - (P<0.05), **Values significantly different between groups (P=<0.01). ***Values significantly different between groups (P=<0.001).

Baseline dietary intakes were comparable between groups. In the LGI group, mean percentage of calories from carbohydrate, amount of carbohydrates, diet GI and GL decreased significantly from baseline, while percentage of energy from protein and dietary fibre increased significantly. The only significant dietary change from baseline in the CHDR group was the reduction in the percentage of energy from fat. After six months, mean percentage of calories from carbohydrate, GI and GL were significantly lower in the LGI compared to the CHDR group. Fibre intake was significantly higher in the LGI group. During the study period, only changes in dietary glycaemic index and dietary fibre intakes were significantly different between groups (Refer Table 5).

Based on the results from regression analysis, mean GL during the six months after intervention was the single independent predictor of weight loss in subjects (R^2^= 13.5%, standardized B= 0.37, P= 0.003). Weight change was the single independent predictor of changes in 2HPP (R^2^= 7.4%, standardized B= 0.272, P= 0.02). Change in 2HPP was the independent predictor of change in FBS (R^2^=32. 7%, standardized B= 0.572 P< 0.001).

In subjects with INS≥2 μIU/L, mean GI primarily explained changes in 2HPP mean (R^2^=43. 8%, standardized B= −0.662 P< 0.001), with reduction in 2HPP being caused by a reduction in GI. Further analysis with block regression showed that mean GI explained 43.8% of the change in 2HPP while weight change explained only an additional of 3.9% (P=0.227) in these subjects. The Sobel’s method was used to further determine if the effect of GI on 2HPP was mediated through changes in weight. The results showed that mediation effect was small and not significant in subjects with INS≥2 μIU/L (Indirect effect vs. Direct effect: unstandardized B= −0.05 vs. 0.196, P values 0.716 vs. <0.001).

### Physical activity and adherence

Physical activity levels remained comparable between the groups at baseline (LGI vs. CHDR: median (IQR):608 (2727) vs. 900(2304) MET.min/week, P=0.143) and at the end of six months study period (933 (1403) vs.965 (857) MET.min/week, P= 0.908). Dietary adherence (self reported and calculated, data not shown here) were statistically comparable between the groups.

## Discussion

The current study was designed to investigate the effects of adding low-GI nutrition education to conventional healthy dietary advice in subjects with previous history of GDM. Baseline characteristics were well matched in terms of socio-demographic and metabolic variables. Subjects in the LGI group of this study lost an average of 1.3 kg compared to the 0.1 kg loss in the CHDR arm, after six months on the trial. These findings are in agreement with the Cochrane review which concluded that subjects on low GI diets lost an average of 1 kg more than subjects on high GI diets, in trials that lasted up to 6 months in duration [40]. In this study, more post-GDM subjects in LGI group achieved a moderate 5% weight loss (P=0.01). Hence, low GI diets aid weight loss in the study population of women with previous GDM as compared to conventional healthy diets with similar energy prescriptions, during a 6 month period.

Moderate weight loss in the range of 5-10% has been proven to improve cardio-metabolic risk in high risk subjects [41]. Furthermore, every 1 kg weight reduction achieved in the first year of the Diabetes Prevention Program (DPP) translated into a 16% reduction in risk for conversion to T2DM [42]. Hence by promoting moderate weight loss in the group of post-GDM subjects, LGI diets may contribute effectively to lowering their cardio-metabolic risks.

CHDR subjects had a significant increase of 0.6 mmol/L from the baseline 2HPP levels, after six months’ intervention. The magnitude of this change described by its effect size is “moderate” and remains a cause for concern in GDM women who are known to be at an increased risk for development of glucose intolerance [3]. Therefore, conventional healthy diets that emphasize on calorie and fat control do not benefit these women with prior GDM, in terms of improving glucose tolerance.

The LGI group, on the contrary, had maintained their 2HPP levels during the same period of study. As an increase in 2HPP predominantly represents glucose intolerance [17], lowering of 2HPP within these subjects by LGI diet would lead to the reduction of the prevalence of abnormal glucose tolerance. This line of thought finds further support in the higher rate of reversal to normoglycaemia observed among IGT subjects in LGI group after six months (50% vs. 40%, P= 0.689.). Furthermore, post-challenge plasma glucose spikes are thought to be more strongly associated with risk for atherosclerosis than FBS in a cohort at risk for diabetes [43]. Hence, the improvement in glucose tolerance could delay or prevent the subsequent development of T2DM and its complications in this high risk group of subjects.

There is a paucity of data on the normal fasting insulin range for young healthy Malaysian women. A Malaysian study among 30 healthy volunteers reported a median fasting insulin level of 4.7 μIU/ml with a central 95% range of 2.1 to 12.1 μIU/ml [44]. Baseline INS in 18/38 subjects in CHDR group and 15/39 subjects in LGI group were <2 μIU/ml. Given that women with prior GDM have innate tendencies towards insulin resistance [45], one would expect our study population to have higher fasting insulin levels. It was unexpected that 57% of our subjects would have INS less than the 2.1 μIU/ml. However, Farhan et al., (2012) also found the mean fasting insulin values, of a small group (n=10) of Austrian GDM women, at 3 month postpartum to be 1.63 μIU/ml. One possible explanation for this occurrence is that only 43% of our subjects could be classified as being obese (BMI>27.5) [46], which could have resulted in the normoglycaemic state represented in the fasting blood glucose and normal or low INS. The median BMI of this study group was 26, and hence these subjects could be defined only as being mildly overweight based on the WHO criteria [47]. This finding suggests that despite their inherent insulin resistance accentuated by pregnancy, these subjects were at a very early stage of pre-diabetes, making early detection and intervention necessary if not paramount. We acknowledge that a large cross sectional study of healthy Malaysian individuals is necessary to determine normal reference fasting insulin range for this population.

The dietary intervention resulted in significant different outcomes in subjects with baseline INS≥2 μIU/ml with respect to anthropometry and 2HPP. For subjects with baseline INS≥2 μIU/ml the greatest improvement in glucose tolerance was noted among subjects in the lowest quartile for dietary GI at 6 months. Furthermore these subjects also achieved more weight loss when on LGI diets. These findings are consistent with the 6 months finding from the CALERIE study which reported that women with higher postprandial insulinaemic response lost more weight on low GI diets after 6 months on intervention [14]. However the mean baseline fasting insulin levels in the CALERIE subjects was 11.9 μIU/ml, which was much higher than the mean for this study group.

Additionally, among subjects with higher baseline INS, the LGI group observed a significant reduction in the 2HPP while the 2HPP levels increased in the CHDR group. This difference in 2HPP changes between groups was statistically significant (Mean ∆(LGI-CHDR) =2.4 mmol/L, P=0.004). Since interventions that reduced 2-h plasma glucose by >0.84 mmol/L are thought to halve the risk for incidence of T2DM [36], LGI diets may be highly recommended for post-GDM women with higher fasting insulin levels. Furthermore, in subjects with INS≥2 μIU/L the reduction in 2HPP brought about by lowering dietary GI was not primarily mediated through body weight loss. This mechanism of action is of special interest in post-GDM women, who have little success in attaining moderate weight loss compared to other high risk groups [1].

The favourable anthropometric and glycaemic responses to low GI intervention in those with higher insulin levels (as against low or normal insulin secretors) can be explained by the exaggerated responses to increase in GI demonstrated among hyperinsulinaemic (insulin resistant) subjects [14]. While we do not possess the postprandial insulin values for our subjects to classify them as having typical hyperinsulinaemic response to a glucose challenge, an increase in fasting insulin concentrations is also associated with lower liver insulin clearance, which conserves insulin and contributes to hyperinsulinaemia [48]. Recent studies further corroborate the findings that dietary GI may have varying effects depending on individual metabolic functioning of the “adipo-insular axis” [49].

A trend for increasing weight and 2HPP blood glucose was observed in subjects with dysglycaemia within the CHDR group. Furthermore a higher likelihood of conversion to normoglycaemia among dysglycaemic subjects was observed in the LGI group. In the current trial, 37.5% and 64.3% of subjects with dysglycaemia at baseline, returned to normoglycaemia at 6 months in CHDR and LGI groups respectively. The percentage of dysglycaemic subjects returning to normoglycaemia in the LGI arm of our study was comparable to the results from lifestyle intervention in China that also used acarbose and metformin [50]. Hence, LGI diets could be more effective in managing glycaemia among hyperinsulinaemic, dysglycaemic women with a previous history of GDM.

We acknowledge the following limitations to the study. This study is limited by the fact that the dietary intake including GI and GL were calculated based on reported intakes, though attempts were made to ensure completeness of reporting. The 3-day food record was chosen to evaluate dietary data. This was done so since this tool is the least intrusive of diet recording techniques and was hence best suited to this group of subjects with many recognized barriers to participate in preventive interventions [5]. To improve the accuracy of the reporting, subjects were trained to use the three day food records at the screening visit and assisted by illustrations of household measures to aid in recording amounts consumed. Secondly, fifty- two percent of the subjects were identified as under-reporters based on a EI:BMR<1.2, [51]) at baseline. However, these observations were within the estimates for prevalence of under-reporting among overweight and obese women [52]. Furthermore, the percentage of under-reporters in LGI and CHDR groups were comparable between groups (p=0.405). Furthermore, excluding the under-reporters did not alter the statistical significance observed in the dietary intake among subjects. Food records also do not capture details of food processing and other factors affecting dietary variables, including GI. Nevertheless, outcome changes in the LGI and CHDR groups were consistent with previous reports for similar dietary intake comparisons [34,35], thereby adding credence to the dietary data obtained from the subjects.

Self-reported dietary data are considered acceptable as indicators of dietary quality including percentage contribution to energy from macronutrients [53]. Dietary GI, being a measure of quality could also be expected to be minimally affected by underreporting. However, it is felt that underreporting may have also contributed to the smaller differences in GL observed between the groups.

It is also acknowledged that the clinically significant 10 point difference in dietary GI between the groups [54], could not be achieved after 6 months of intervention. Also the dietary GI for the LGI group was actually in the intermediate GI category (diet GI less than sixty [16]). However, more subjects in LGI had intermediate GI (62 vs. 21%), while more subjects in the CHDR group were in the high GI category (79 vs. 37%). Asian trials that achieved comparable difference in GI of around six units between groups, have also documented significant beneficial effects in terms of reductions in waist circumference, fasting blood sugar and glycaemic control in diabetic subjects or those with impaired fasting glucose [34,35]. This study was able to demonstrate that moderate reductions in GI of seven units had beneficial effects on the anthropometric and 2HPP outcomes of Asian post-GDM women with postpartum normoglycaemia, IFG or IGT.

We concede that neither group reached the universal 25-30 g dietary fibre intake recommendation. Achieving this target in the Malaysian environment has been recognized as a challenge [55]. However, it should be noted that LGI group had a significantly higher fibre intake as compared to the CHDR group in this study (17 vs. 13 g, P<0.001). Thus, adding GI education to CHDR improved dietary fibre intakes in subjects which could not be achieved with CHDR. Such additional benefits in dietary quality when using low GI diets in the Asian population has also been documented earlier [56].

We do not possess formal data on satisfaction of the subjects with the two dietary interventions. However, self-reported adherence and calculated adherence to energy and fat intake prescriptions and drop-out rates were comparable between the groups. Thus it may be appropriate to consider that acceptance to both interventions may also be similar.

The assay employed to measure INS samples in this study, failed to analyse samples with INS <2 μIU/ml. Hence the INS cut-off used to group subjects to conduct the sub-analysis to investigate the differential effect of the diets in subjects with varying insulin levels can be considered arbitrary. However, the varied metabolic response to the two diets provides a strong evidence for the added benefit of lowering GI for subjects with higher insulin levels.

## Conclusions

This six months intervention study showed that lowering the dietary GI of conventional diets improved glucose tolerance and reduced body weight, in comparison to the conventional diet in women with previous history of GDM. Subjects with higher fasting insulin level in the low GI group demonstrated the most positive effects in the improvement of their dysglycaemia and weight loss.

## Abbreviations

2HPP: 2-h post 75 g OGTT blood glucose; BMR: Basal metabolic rate; FBS: Fasting blood sugar; GDM: Gestational diabetes mellitus; GI: Glycaemic index; GL: Glycaemic load; INS: Fasting serum insulin; OGTT: Oral glucose tolerance test; T2DM: Type 2 diabetes mellitus.

## Competing interests

The authors have no potential conflict of interests to declare.

## Authors’ contributions

NAK and FA conceived the project, designed the study, and were involved in data interpretation and editing of the manuscript. SS and RAG helped with study design, data collection, data analysis, interpretation and wrote the manuscript. NAW was involved in study design, data collection, and editing the manuscript. BNMY and NSS participated in designing the study, data interpretation and editing of the manuscript. KC was involved in study design, data analysis and interpretation and editing of the manuscript. All authors read and approved the final manuscript.
